# Using a theory of mind to find best responses to memory-one strategies

**DOI:** 10.1038/s41598-020-74181-y

**Published:** 2020-10-14

**Authors:** Nikoleta E. Glynatsi, Vincent A. Knight

**Affiliations:** 1 School of Mathematics, Cardiff University, Cardiff, CF24 4AG UK; 2 Max Planck Institute for Evolutionary Biology, Plön, 24 306 Germany

**Keywords:** Evolution, Mathematics and computing

## Abstract

Memory-one strategies are a set of Iterated Prisoner’s Dilemma strategies that have been praised for their mathematical tractability and performance against single opponents. This manuscript investigates *best response* memory-one strategies with a theory of mind for their opponents. The results add to the literature that has shown that extortionate play is not always optimal by showing that optimal play is often not extortionate. They also provide evidence that memory-one strategies suffer from their limited memory in multi agent interactions and can be out performed by optimised strategies with longer memory. We have developed a theory that has allowed to explore the entire space of memory-one strategies. The framework presented is suitable to study memory-one strategies in the Prisoner’s Dilemma, but also in evolutionary processes such as the Moran process. Furthermore, results on the stability of defection in populations of memory-one strategies are also obtained.

## Introduction

The Prisoner’s Dilemma (PD) is a two player game used in understanding the evolution of cooperative behaviour, formally introduced in^1^. Each player has two options, to cooperate (C) or to defect (D). The decisions are made simultaneously and independently. The normal form representation of the game is given by:

1 $$\begin{aligned} S_p = \begin{pmatrix} R &{} S \\ T &{} P \end{pmatrix} \quad S_q = \begin{pmatrix} R &{} T \\ S &{} P \end{pmatrix} \end{aligned}$$

where $S_p$ represents the utilities of the row player and $S_q$ the utilities of the column player. The payoffs, $(R, P, S, T)$, are constrained by $T> R> P > S$ and $2R > T + S$, and the most common values used in the literature are $(R, P, S, T) = (3, 1, 0, 5)$^2^. The numerical experiments of our manuscript are carried out using these payoff values. The PD is a one shot game, however, it is commonly studied in a manner where the history of the interactions matters. The repeated form of the game is called the Iterated Prisoner’s Dilemma (IPD).

Memory-one strategies are a set of IPD strategies that have been studied thoroughly in the literature^3,4^, however, they have gained most of their attention when a certain subset of memory-one strategies was introduced in^5^, the zero-determinant strategies (ZDs). In^6^ it was stated that “Press and Dyson have fundamentally changed the viewpoint on the Prisoner’s Dilemma”. A special case of ZDs are extortionate strategies that choose their actions so that a linear relationship is forced between the players’ score ensuring that they will always receive at least as much as their opponents. ZDs are indeed mathematically unique and are proven to be robust in pairwise interactions, however, their true effectiveness in tournaments and evolutionary dynamics has been questioned^7–12^.

The purpose of this work is to reinforce the literature on the limitations of extortionate strategies by considering a new approach. More specifically, by considering best response memory-one strategies with a theory of mind of their opponents. There are several works in the literature that have considered strategies with a theory of mind^5,6,13–16^. These works defined “theory of mind” as intention recognition^13–16^ and as the ability of a strategy to realise that their actions can influence opponents^6^. Compared to these works, theory of mind is defined here as the ability of a strategy to know the behaviour of their opponents and alter their own behaviour in response to that.

We present a closed form algebraic expression for the utility of a memory-one strategy against a given set of opponents and a compact method of identifying it’s best response to that given set of opponents. The aim is to evaluate whether a best response memory-one strategy behaves in a zero-determinant way which in turn indicates whether it can be extortionate. We do this using a linear algebraic approach presented in^17^. This is done in tournaments with two opponents. Moreover, we introduce a framework that allows the comparison of an optimal memory-one strategy and an optimised strategy which has a larger memory.

To illustrate the analytical results obtained in this manuscript a number of numerical experiments are run. The source code for these experiments has been written in a sustainable manner^18^. It is open source (https://github.com/Nikoleta-v3/Memory-size-in-the-prisoners-dilemma) and tested which ensures the validity of the results. It has also been archived and can be found at^19^.

## Methods

One specific advantage of memory-one strategies is their mathematical tractability. They can be represented completely as an element of ${\mathbb {R}}^{4}_{[0, 1]}$. This originates from^20^ where it is stated that if a strategy is concerned with only the outcome of a single turn then there are four possible ‘states’ the strategy could be in; both players cooperated ($CC$), the first player cooperated whilst the second player defected ($CD$), the first player defected whilst the second player cooperated ($DC$) and both players defected ($DD$). Therefore, a memory-one strategy can be denoted by the probability vector of cooperating after each of these states; $p=(p_1, p_2, p_3, p_4) \in {\mathbb {R}}_{[0,1]} ^ 4$.

In^20^ it was shown that it is not necessary to simulate the play of a strategy *p* against a memory-one opponent *q*. Rather this exact behaviour can be modeled as a stochastic process, and more specifically as a Markov chain whose corresponding transition matrix $M$ is given by Eq. (2). The long run steady state probability vector $v$, which is the solution to $v M = v$, can be combined with the payoff matrices of Eq. (1) to give the expected payoffs for each player. More specifically, the utility for a memory-one strategy $p$ against an opponent $q$, denoted as $u_q(p)$, is given by Eq. (3).

2 $$\begin{aligned} M= & {} \left[ \begin{matrix}p_{1} q_{1} &{} p_{1} \left( - q_{1} + 1\right) &{} q_{1} \left( - p_{1} + 1\right) &{} \left( - p_{1} + 1\right) \left( - q_{1} + 1\right) \\ p_{2} q_{3} &{} p_{2} \left( - q_{3} + 1\right) &{} q_{3} \left( - p_{2} + 1\right) &{} \left( - p_{2} + 1\right) \left( - q_{3} + 1\right) \\ p_{3} q_{2} &{} p_{3} \left( - q_{2} + 1\right) &{} q_{2} \left( - p_{3} + 1\right) &{} \left( - p_{3} + 1\right) \left( - q_{2} + 1\right) \\ p_{4} q_{4} &{} p_{4} \left( - q_{4} + 1\right) &{} q_{4} \left( - p_{4} + 1\right) &{} \left( - p_{4} + 1\right) \left( - q_{4} + 1\right) \end{matrix}\right] \end{aligned}$$

3 $$\begin{aligned} u_q(p)= & {} v \cdot (R, S, T, P). \end{aligned}$$

This manuscript has explored the form of $u_q(p)$, to the best of the authors knowledge no previous work has done this, and Theorem 1 states that $u_q(p)$ is given by a ratio of two quadratic forms^21^.

### Theorem 1

*The expected utility of a memory-one strategy* $p\in {\mathbb {R}}_{[0,1]}^4$ *against a memory-one opponent* $q\in {\mathbb {R}}_{[0,1]}^4$, *denoted as* $u_q(p)$, *can be written as a ratio of two quadratic forms:*

4 $$\begin{aligned} u_q(p) = \frac{\frac{1}{2}pQp^T + cp + a}{\frac{1}{2}p{\bar{Q}}p^T + {\bar{c}}p + {\bar{a}}}, \end{aligned}$$

*where* $Q, {\bar{Q}}$ $\in {\mathbb {R}}^{4\times 4}$ *are square matrices defined by the transition probabilities of the opponent* $q_1, q_2, q_3, q_4$ *as follows:*

5 $$\begin{aligned} Q= & {} \left[ \begin{matrix}0 &{} - \left( q_{1} - q_{3}\right) \left( P q_{2} - P - T q_{4}\right) &{} \left( q_{1} - q_{2}\right) \left( P q_{3} - S q_{4}\right) &{} \left( q_{1} - q_{4}\right) \left( S q_{2} - S - T q_{3}\right) \\ - \left( q_{1} - q_{3}\right) \left( P q_{2} - P - T q_{4}\right) &{} 0 &{} \left( q_{2} - q_{3}\right) \left( P q_{1} - P - R q_{4}\right) &{} - \left( q_{3} - q_{4}\right) \left( R q_{2} - R - T q_{1} + T\right) \\ \left( q_{1} - q_{2}\right) \left( P q_{3} - S q_{4}\right) &{} \left( q_{2} - q_{3}\right) \left( P q_{1} - P - R q_{4}\right) &{} 0 &{} \left( q_{2} - q_{4}\right) \left( R q_{3} - S q_{1} + S\right) \\ \left( q_{1} - q_{4}\right) \left( S q_{2} - S - T q_{3}\right) &{} - \left( q_{3} - q_{4}\right) \left( R q_{2} - R - T q_{1} + T\right) &{} \left( q_{2} - q_{4}\right) \left( R q_{3} - S q_{1} + S\right) &{} 0\end{matrix}\right] , \end{aligned}$$

6 $$\begin{aligned} {\bar{Q}}= & {} \left[ \begin{matrix}0 &{} - \left( q_{1} - q_{3}\right) \left( q_{2} - q_{4} - 1\right) &{} \left( q_{1} - q_{2}\right) \left( q_{3} - q_{4}\right) &{} \left( q_{1} - q_{4}\right) \left( q_{2} - q_{3} - 1\right) \\ - \left( q_{1} - q_{3}\right) \left( q_{2} - q_{4} - 1\right) &{} 0 &{} \left( q_{2} - q_{3}\right) \left( q_{1} - q_{4} - 1\right) &{} \left( q_{1} - q_{2}\right) \left( q_{3} - q_{4}\right) \\ \left( q_{1} - q_{2}\right) \left( q_{3} - q_{4}\right) &{} \left( q_{2} - q_{3}\right) \left( q_{1} - q_{4} - 1\right) &{} 0 &{} - \left( q_{2} - q_{4}\right) \left( q_{1} - q_{3} - 1\right) \\ \left( q_{1} - q_{4}\right) \left( q_{2} - q_{3} - 1\right) &{} \left( q_{1} - q_{2}\right) \left( q_{3} - q_{4}\right) &{} - \left( q_{2} - q_{4}\right) \left( q_{1} - q_{3} - 1\right) &{} 0\end{matrix}\right] . \end{aligned}$$

$c \text { and } {\bar{c}}$ $\in {\mathbb {R}}^{4 \times 1}$ *are similarly defined by:*

7 $$\begin{aligned} c= & {} \left[ \begin{matrix}q_{1} \left( P q_{2} - P - T q_{4}\right) \\ - \left( q_{3} - 1\right) \left( P q_{2} - P - T q_{4}\right) \\ - P q_{1} q_{2} + P q_{2} q_{3} + P q_{2} - P q_{3} + R q_{2} q_{4} - S q_{2} q_{4} + S q_{4}\\ - R q_{2} q_{4} + R q_{4} + S q_{2} q_{4} - S q_{2} - S q_{4} + S + T q_{1} q_{4} - T q_{3} q_{4} + T q_{3} - T q_{4}\end{matrix}\right] , \end{aligned}$$

8 $$\begin{aligned} {\bar{c}}= & {} \left[ \begin{matrix}q_{1} \left( q_{2} - q_{4} - 1\right) \\ - \left( q_{3} - 1\right) \left( q_{2} - q_{4} - 1\right) \\ - q_{1} q_{2} + q_{2} q_{3} + q_{2} - q_{3} + q_{4}\\ q_{1} q_{4} - q_{2} - q_{3} q_{4} + q_{3} - q_{4} + 1\end{matrix}\right] , \end{aligned}$$

*and the constant terms* $a, {\bar{a}}$ *are defined as* $a = - P q_{2} + P + T q_{4}$ and ${\bar{a}} = - q_{2} + q_{4} + 1\mathbf{}$.

The proof of Theorem 1 is given in the Supplementary Information. Theorem 1 can be extended to consider multiple opponents. The IPD is commonly studied in tournaments and/or Moran Processes where a strategy interacts with a number of opponents. The payoff of a player in such interactions is given by the average payoff the player received against each opponent. More specifically the expected utility of a memory-one strategy against $N$ opponents is given by:

9 $$\begin{aligned}&\frac{1}{N} \sum \limits _{i=1} ^ {N} {u_q}^{(i)} (p) = \frac{\frac{1}{N} \sum \limits _{i=1} ^ {N} (\frac{1}{2} pQ^{(i)} p^T + c^{(i)} p + a^ {(i)}) \prod \limits _{\tiny \begin{array}{l} j=1 \\ j \ne i \end{array}} ^ N (\frac{1}{2} p{\bar{Q}}^{(j)} p^T + {\bar{c}}^{(j)} p + {\bar{a}}^ {(j)})}{\prod \limits _{i=1} ^ N (\frac{1}{2} p{\bar{Q}}^{(i)} p^T + {\bar{c}}^{(i)} p + {\bar{a}}^ {(i)})}. \end{aligned}$$

Equation (9) is the average score (using Eq. (4)) against the set of opponents.

Estimating the utility of a memory-one strategy against any number of opponents without simulating the interactions is the main result used in the rest of this manuscript. It will be used to obtain best response memory-one strategies in tournaments in order to explore the limitations of extortion and restricted memory.

## Results

The formulation as presented in Theorem 1 can be used to define *memory-one best response* strategies as a multi dimensional optimisation problem given by:

10 $$\begin{aligned} \begin{aligned} \max _p:&\ \sum _{i=1} ^ {N} {u_q}^{(i)} (p)\\ \text {such that}:&\ p \in {\mathbb {R}}_{[0, 1]} \end{aligned} \end{aligned}$$

Optimising this particular ratio of quadratic forms is not trivial. It can be verified empirically for the case of a single opponent that there exists at least one point for which the definition of concavity does not hold. The non concavity of $u(p)$ indicates multiple local optimal points. This is also intuitive. The best response against a cooperator, $q=(1, 1, 1, 1)$, is a defector $p^*=(0, 0, 0, 0)$. The strategies $p=\left( \frac{1}{2}, 0, 0, 0\right)$ and $p=\left( \frac{1}{2}, 0, 0, \frac{1}{2}\right)$ are also best responses. The approach taken here is to introduce a compact way of constructing the discrete candidate set of all local optimal points, and evaluating the objective function Eq. (9). This gives the best response memory-one strategy. The approach is given in Theorem 2.

### Theorem 2

*The optimal behaviour of a memory-one strategy player* $p^* \in {\mathbb {R}}_{[0, 1]} ^ 4$ *against a set of* $N$ *opponents* $\{q^{(1)}, q^{(2)}, \dots , q^{(N)} \}$ for $q^{(i)} \in {\mathbb {R}}_{[0, 1]} ^ 4$ is *given by:*

$$\begin{aligned} p^* = \text {argmax}\sum \limits _{i=1} ^ N u_q(p), \ p \in S_q. \end{aligned}$$

*The set* $S_q$ *is defined as all the possible combinations of:*

11 $$ \begin{aligned} S_q = \left\{ p \in {\mathbb {R}} ^ 4 \left| \begin{aligned} \bullet \quad p_j \in \{0, 1\}&\quad \text {and} \quad \frac{d}{dp_k} \sum \limits _{i=1} ^ N u_q^{(i)}(p) = 0 \\&\quad \text {for all} \quad j \in J \quad  \&  \quad k \in K \quad \text {for all} \quad J, K \\&\quad \text {where} \quad J \cap K = \emptyset \quad \text {and} \quad J \cup K = \{1, 2, 3, 4\}.\\ \bullet \quad p \in \{0, 1\} ^ 4 \end{aligned}\right. \right\} . \end{aligned}$$

*Note that there is no immediate way to find the zeros of* $\frac{d}{dp} \sum \limits _{i=1} ^ N u_q(p)$ *where,*

12 $$\begin{aligned} \frac{d}{dp} \sum \limits _{i=1} ^ {N} {u_q}^{(i)} (p)&= \displaystyle \sum \limits _{i=1} ^ {N} \frac{\left( pQ^{(i)} + c^{(i)}\right) \left( \frac{1}{2} p{\bar{Q}}^{(i)} p^T + {\bar{c}}^{(i)} p + {\bar{a}}^ {(i)}\right) }{\left( \frac{1}{2} p{\bar{Q}}^{(i)} p^T + {\bar{c}}^{(i)} p + {\bar{a}}^ {(i)}\right) ^ 2} - \frac{\left( p{\bar{Q}}^{(i)} + {\bar{c}}^{(i)}\right) \left( \frac{1}{2} pQ^{(i)} p^T + c^{(i)} p + a^ {(i)}\right) }{\left( \frac{1}{2} p{\bar{Q}}^{(i)} p^T + {\bar{c}}^{(i)} p + {\bar{a}}^ {(i)}\right) ^ 2} \end{aligned}$$

*For* $\frac{d}{dp} \sum \limits _{i=1} ^ N u_q(p)$ *to equal zero then:*

13 $$\begin{aligned}&\displaystyle \sum \limits _{i=1} ^ {N} \left( pQ^{(i)} + c^{(i)}\right) \left( \frac{1}{2} p{\bar{Q}}^{(i)} p^T + {\bar{c}}^{(i)} p + {\bar{a}}^ {(i)}\right) - \left( p{\bar{Q}}^{(i)} + {\bar{c}}^{(i)}\right) \left( \frac{1}{2} pQ^{(i)} p^T + c^{(i)} p + a^ {(i)}\right) \quad = 0, \quad {while} \end{aligned}$$

14 $$\begin{aligned}&\displaystyle \sum \limits _{i=1} ^ {N} \frac{1}{2} p{\bar{Q}}^{(i)} p^T + {\bar{c}}^{(i)} p + {\bar{a}}^ {(i)} \ne 0. \end{aligned}$$

The proof of Theorem 2 is given in the Supplementary Information. Finding best response memory-one strategies is analytically feasible using the formulation of Theorem 2 and resultant theory^22^. However, for large systems building the resultant becomes intractable. As a result, best responses will be estimated heuristically using a numerical method, suitable for problems with local optima, called Bayesian optimisation^23^.

### Limitations of extortionate behaviour

In multi opponent settings, where the payoffs matter, strategies trying to exploit their opponents will suffer. Compared to ZDs, best response memory-one strategies, which have a theory of mind of their opponents, utilise their behaviour in order to gain the most from their interactions. The question that arises then is whether best response strategies are optimal because they behave in an extortionate way.

The results of this section use Bayesian optimisation to generate a data set of best response memory-one strategies for $N=2$ opponents. The data set is available at^24^. It contains a total of 1000 trials corresponding to 1000 different instances of a best response strategy in tournaments with $N=2$. For each trial a set of 2 opponents is randomly generated and the memory-one best response against them is found. In order to investigate whether best responses behave in an extortionate matter the SSE method described in^17^ is used. More specifically, in^17^ the point $x^*$, in the space of memory-one strategies, that is the nearest extortionate strategy to a given strategy $p$ is given by,

15 $$\begin{aligned} x^* = {\left( C^{T}C\right) }^{-1}C^{T}{\bar{p}} \end{aligned}$$

where $${\bar{p}}=(p_1 - 1, p_2 - 1, p_3, p_4)$$ and

16 $$\begin{aligned} C = \begin{bmatrix} R - P &{} R- P \\ S - P &{} T- P \\ T - P &{} S- P \\ 0 &{} 0 \\ \end{bmatrix}. \end{aligned}$$

Once this closest ZDs is found, the squared norm of the remaining error is referred to as sum of squared errors of prediction (SSE):

17 $$\begin{aligned} \text {SSE} = {{\bar{p}}} ^ T {\bar{p}} - {\bar{p}} C \left( C ^ T C \right) ^ {-1} C ^ T {\bar{p}} = {{\bar{p}}} ^ T {\bar{p}} - {\bar{p}} C x ^ * \end{aligned}$$

**Figure 1:**
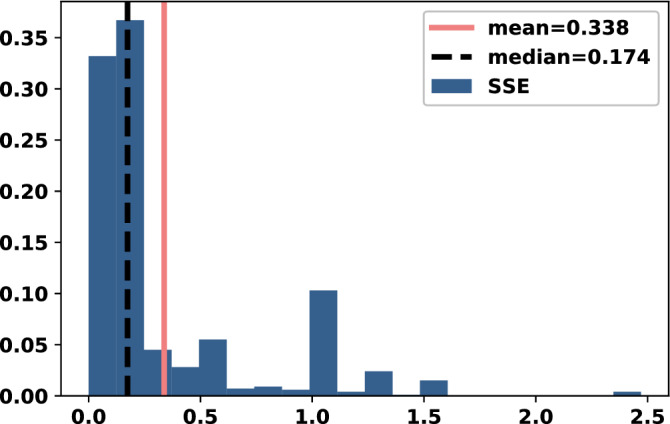
SEE distribution for best response in tournaments with $N=2$.

Thus, SSE is defined as how far a strategy is from behaving as a ZD. A high SSE implies a non extortionate behaviour. The distribution of SSE for the best response in tournaments ($N=2$) is given in Fig. 1. Moreover, a statistical summary of the SSE distribution is given in Table 1.

**Table 1:** SSE of best response memory-one when $N=2$.

Mean	Std	5%	50%	95%	Max	Median	Skew	Kurt
0.34	0.40	0.028	0.17	1.05	2.47	0.17	1.87	3.60

For the best response in tournaments with $N=2$ the distribution of SSE is skewed to the left, indicating that the best response does exhibit ZDs behaviour and so could be extortionate, however, the best response is not uniformly a ZDs. A positive measure of skewness and kurtosis, and a mean of 0.34 indicate a heavy tail to the right. Therefore, in several cases the strategy is not trying to extort its opponents. This highlights the importance of adaptability since the best response strategy against an opponent is rarely (if ever) a unique ZDs.

### Limitations of memory size

The other main finding presented in^5^ was that short memory of the strategies was all that was needed. We argue that the second limitation of ZDs in multi opponent interactions is that of their restricted memory. To demonstrate the effectiveness of memory in the IPD we explore a best response longer-memory strategy against a given set of memory-one opponents, and compare it’s performance to that of a memory-one best response.

In^25^, a strategy called *Gambler* which makes probabilistic decisions based on the opponent’s $n_1$ first moves, the opponent’s $m_1$ last moves and the player’s $m_2$ last moves was introduced. In this manuscript Gambler with parameters: $n_1 = 2, m_1 = 1$ and $m_2 = 1$ is used as a longer-memory strategy. By considering the opponent’s first two moves, the opponents last move and the player’s last move, there are only 16 $(4 \times 2 \times 2)$ possible outcomes that can occur, furthermore, Gambler also makes a probabilistic decision of cooperating in the opening move. Thus, Gambler is a function $f: \{\text {C, D}\} \rightarrow [0, 1]_{{\mathbb {R}}}$. This can be hard coded as an element of $[0, 1]_{{\mathbb {R}}} ^ {16 + 1}$, one probability for each outcome plus the opening move. Hence, compared to Eq. (10), finding an optimal Gambler is a 17 dimensional problem given by:

18 $$\begin{aligned} \begin{aligned} \max _p:&\ \sum _{i=1} ^ {N} {U_q}^{(i)} (f) \\ \text {such that}:&\ f \in {\mathbb {R}}_{[0, 1]}^{17} \end{aligned} \end{aligned}$$

Note that Eq. (9) can not be used here for the utility of Gambler, and actual simulated players are used. This is done using^26^ with 500 turns and 200 repetitions, moreover, Eq. (18) is solved numerically using Bayesian optimisation.

Similarly to the previous section, a large data set has been generated with instances of an optimal Gambler and a memory-one best response, available at^24^. Estimating a best response Gambler (17 dimensions) is computational more expensive compared to a best response memory-one (4 dimensions). As a result, the analysis of this section is based on a total of 152 trials. As before, for each trial $N=2$ random opponents have been selected.

**Figure 2:**
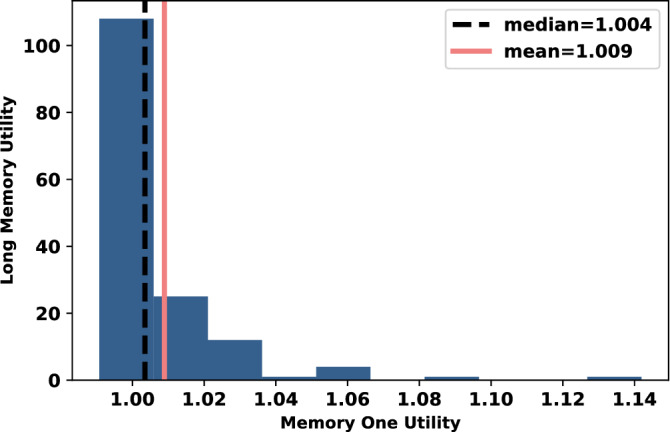
The ratio between the utilities of Gambler and best response memory-one strategy for 152 different pair of opponents.

The ratio between Gambler’s utility and the best response memory-one strategy’s utility has been calculated and its distribution in given in Fig. 2. It is evident from Fig. 2 that Gambler always performs as well as the best response memory-one strategy and often performs better. There are no points where the ratio value is less than 1, thus Gambler never performed less than the best response memory-one strategy and in places outperforms it. However, against two memory-one opponents Gambler’s performance is better than the optimal memory-one strategy. This is evidence that in the case of multiple opponents, having a shorter memory is limiting.

### Dynamic best response player

In several evolutionary settings such as Moran Processes self interactions are key. Previous work has identified interesting results such as the appearance of self recognition mechanisms when training strategies using evolutionary algorithms in Moran processes^11^. This aspect of reinforcement learning can be done for best response memory-one strategies, as presented in this manuscript, by incorporating the strategy itself in the objective function as shown in Eq. (10). Where $K$ is the number of self interactions that will take place.

19 $$\begin{aligned} \begin{aligned} \max _p:&\ \frac{1}{N} \sum \limits _{i=1} ^ {N} {u_q}^{(i)} (p) + Ku_p(p) \\ \text {such that}:&\ p \in {\mathbb {R}}_{[0, 1]} \end{aligned} \end{aligned}$$

For determining the memory-one best response with self interactions, an algorithmic approach called *best response dynamics* is proposed. The best response dynamics approach used in this manuscript is given by Algorithm 1.

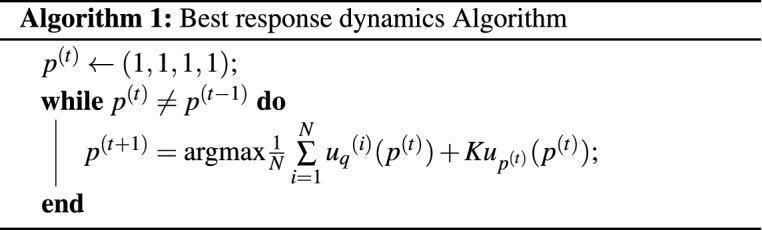



To investigate the effectiveness of this approach, more formally a Moran process will be considered. If a population of $n$ total individuals of two types is considered, with $K$ individuals of the first type and $n-K$ of the second type. The probability that the individuals of the first type will take over the population (the fixation probability) is denoted by $x_K$ and is known to be^27^:

$$\begin{aligned} x_K = \frac{ 1 + \sum _{j=1}^{K-1}\prod _{i=1}^j\gamma _i }{ 1 + \sum _{j=1}^{n-1}\prod _{i=1}^j\gamma _i } \end{aligned}$$

where:

$$\begin{aligned} \gamma _i = \frac{ p_{K, K - 1} }{ p_{K, K + 1} }. \end{aligned}$$

To evaluate the formulation proposed here the best response player (taken to be the first type of individual in our population) will be allowed to act dynamically: adjusting their probability vector at every generation. In essence using the theory of mind to find the best response to not only the opponent but also the distribution of the population. Thus for every value of $K$ there is a different best response player.

Considering the dynamic best response player as a vector $p\in {\mathbb {R}}^4_{[0, 1]}$ and the opponent as a vector $q\in {\mathbb {R}}^4_{[0, 1]}$, the transition probabilities depend on the payoff matrix $A ^ {(K)}$ where:

- $A ^ {(K)}_{11}=u_{p}(p)$ is the long run utility of the best response player against itself.
- $A ^ {(K)}_{12}=u_{q}(p)$ is the long run utility of the best response player against the opponent.
- $A ^ {(K)}_{11}=u_{p}(q)$ is the long run utility of the opponent against the best response player.
- $A ^ {(K)}_{11}=u_{q}(q)$ is the long run utility of the opponent against itself.

The matrix $A ^ {(K)}$ is calculated using Eq. (4). For every value of $K$ the best response dynamics algorithm (Algorithm 1) is used to calculate the best response player.

The total utilities/fitnesses for each player can be written down:

$$\begin{aligned} f_1^{(K)}= & {} (K - 1) A_{11}^{(K)} + (n - K)A_{12}^{(K)}\\ f_2^{(K)}= & {} (K) A_{21}^{(K)} + (n - K - 1)A_{22}^{(K)} \end{aligned}$$

where $f_1^{(K)}$ is the fitness of the best response player, and $f_2^{(K)}$ is the fitness of the opponent.

Using this:

$$\begin{aligned} p_{K, K - 1} = \frac{ (n - K)f_2^{(K)} }{ Kf_1^{(K)}+(n - K)f_2^{(K)} } \frac{ K }{ n } \end{aligned}$$

and:

$$\begin{aligned} p_{K, K + 1} = \frac{ Kf_1^{(K)} }{ Kf_1^{(K)}+(n - K)f_2^{(K)} } \frac{ (n - K) }{ n } \end{aligned}$$

which are all that are required to calculate $x_K$.

**Figure 3:**
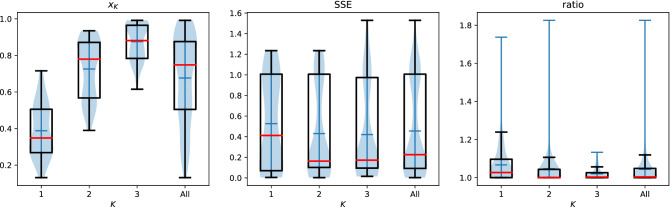
Results for the best response player in a dynamic Moran process. The ratio is taken as the ratio of $x_k$ of the dynamically updating player to the fixation probability of a best response player that does not update as the population density changes.

Figure 3 shows the results of an analysis of $x_K$ for dynamically updating players. This is obtained over 182 Moran process against 122 randomly selected opponents. For each Moran process the fixation probabilities for $K\in \{1, 2, 3\}$ are collected. As well as recording $x_K$, ${\tilde{x}}_K$ is measured where $\tilde{x}_K$ represents the fixation probability of the best response player calculated for a given $K$ but not allowing it to dynamically update as the population changes. The ratio $\frac{x_K}{{\tilde{x}}_K}$ is included in the Figure. This is done to be able to compare to a high performing strategy that has a theory of mind of the opponent but not of the population density. The ratio shows a relatively high performance compared to a non dynamic best response strategy. The mean ratio over all values of $K$ and all experiments is $1.044$. In some cases this dynamic updating results in a 25% increase in the absorption probability.

As denoted before it is clear that the best response strategy in general does not have a low SSE (only 25% of the data is below 0.923 and the average is 0.454) this is further compounded by the ratio being above one showing that in many cases the dynamic strategy benefits from its ability to adapt. This indicates that memory-one strategies that perform well in Moran processes need to more adaptable than a ZDs, and aim for mutual cooperation as well as exploitation which is in line with the results of^28^ where their strategy was designed to adapt and was shown to be evolutionary stable. The findings of this work show that an optimal strategy acts in the same way.

## Discussion

This manuscript has considered *best response* strategies in the IPD game, and more specifically, *memory-one best responses*. It has proven that:

- The utility of a memory-one strategy against a set of memory-one opponents can be written as a sum of ratios of quadratic forms (Theorem 1).
- There is a compact way of identifying a memory-one best response to a group of opponents through a search over a discrete set (Theorem 2).

There is one further theoretical result that can be obtained from Theorem 1, which allows the identification of environments for which cooperation cannot occur (Details are in the Supplementary Information). Moreover, Theorem 2 does not only have game theoretic novelty, but also the mathematical novelty of solving quadratic ratio optimisation problems where the quadratics are non concave.

The empirical results of the manuscript investigated the behaviour of memory-one strategies and their limitations. The empirical results have shown that the performance of memory-one strategies rely on adaptability and not on extortion, and that memory-one strategies’ performance is limited by their memory in cases where they interact with multiple opponents. These relied on two bespoke data sets of 1000 and 152 pairs of memory-one opponents equivalently, archived at^24^.

A further set of results for Moran processes with a dynamically updating best response player was generated and is archived in^29^. This confirmed the previous results which is that high performance requires adaptability and not extortion. It also provides a framework for future stability of optimal behaviour in evolutionary settings.

In the interactions we have considered here the players do not make mistakes; their actions were executed with perfect accuracy. Mistakes, however, are relevant in the reasearch of repeated games^4,30–32^. In future work we would consider interactions with “noise”. Noise can be incoroporated into our formulation and it can be shown that the utility remains a ratio of quadratic forms (Details see the Supplementary Information). Another avenue of investigation would be to understand if and/or when an evolutionary trajectory leads to a best response strategy.

By specifically exploring the entire space of memory-one strategies to identify the best strategy for a variety of situations, this work adds to the literature casting doubt on the effectiveness of ZDs, highlights the importance of adaptability and provides a framework for the continued understanding of these important questions.

## Supplementary information

Supplementary Information.
