# Supplementary material for: Using a theory of mind to find best responses to memory-one strategies
Source: Sci Rep. 2020 Oct 14;10:17287. doi: 10.1038/s41598-020-74181-y (PMC7560663; doi:10.1038/s41598-020-74181-y)
Supplement: Supplementary file 1 — Supplementary Information. [file 41598_2020_74181_MOESM1_ESM.pdf]

# Supplementary Information: Using a theory of mind to find best responses to memory-one strategies.

Nikoleta E. Glynatsi[1,2,*] and Vincent A. Knight[1]

[1]Cardiff University, School of Mathematics, Cardiff, CF24 4AG, United Kingdom
[2]Max Planck Institute for Evolutionary Biology, Plön, 24 306, Germany
[*]glynatsi@evolbio.mpg.de

## 1 Theorem 1 Proof

The utility of a memory one player $p$ against an opponent $q$, $u_q(p)$, can be written as a ratio of two quadratic forms on $R^4$.

*Proof.* It was discussed that $u_q(p)$ it is the product of the steady state vector $v$ and the PD payoffs,

$$u_q(p) = v \cdot (R, S, T, P).$$

The steady state vector which is the solution to $vM = v$ is given by

$$v = \Bigg[\frac{p_2p_3(q_2q_4 - q_3q_4) + p_2p_4(q_2q_3 - q_2q_4 - q_3 + q_4) + p_3p_4(-q_2q_3 + q_3q_4) - p_3q_2q_4 + p_4q_4(q_2 - 1)}{\bar{v}},$$
$$\frac{p_1p_3(q_1q_4 - q_2q_4) + p_1p_4(-q_1q_2 + q_1 + q_2q_4 - q_4) + p_3p_4(q_1q_2 - q_1q_4 - q_2 + q_4) + p_3q_4(q_2 - 1) - p_4q_2(q_4 + 1) + p_4(q_4 - 1)}{\bar{v}},$$
$$\frac{-p_1p_2(q_1q_4 - q_3q_4) - p_1p_4(-q_1q_3 + q_3q_4) + p_1q_1q_4 - p_2p_4(q_1q_3 - q_1q_4 - q_3 + q_4) - p_2q_4(q_3 + 1) - p_4q_4(q_1 + q_3) - p_4(q_3 + q_4) - q_4}{\bar{v}},$$
$$\frac{p_1p_2(q_1q_2 - q_1 - q_2q_3 + q_3) + p_1p_3(-q_1q_3 + q_2q_3) - p_1q_1(q_2 + 1) + p_2p_3(-q_1q_2 + q_1q_3 + q_2 - q_3) + p_2(q_3q_2 - q_2 - q_3 - 1) + p_3(q_1q_2 - q_3q_2 - q_2 - q_3) + q_2 - 1}{\bar{v}}\Bigg],$$

where,

$$\begin{aligned}\bar{v} = \; & p_1p_2(q_1q_2 - q_1q_4 - q_1 - q_2q_3 + q_3q_4 + q_3) - p_1p_3(q_1q_3 - q_1q_4 - q_2q_3 + q_2q_4) - p_1p_4(q_1q_2 - q_1q_3 - q_1 - q_2q_4 + q_3q_4 + q_4) - \\ & p_1q_1(q_2 + q_4 + 1) + p_2p_3(-q_1q_2 + q_1q_3 + q_2q_4 + q_2 - q_3q_4 - q_3) + p_2p_4(-q_1q_3 + q_1q_4 + q_2q_3 - q_2q_4) + p_2q_2(q_3 - 1) - p_2q_3(q_4 - 1) + \\ & p_2(q_4 + 1) + p_3p_4(q_1q_2 - q_1q_4 - q_2q_3 - q_2 + q_3q_4 + q_4) + p_3q_2q_1(-p_3 - 1) + p_3(q_3 - q_4) - p_4(q_1q_4 + q_2 + q_3q_4 - q_3 + q_4 - 1) + \\ & q_2 - q_4 - 1\end{aligned}$$

The dot product of $v \cdot (R, S, T, P)$ gives,

$$u_q(p) = \frac{R\left(p_2p_3(q_2q_4 - q_3q_4) + p_2p_4(q_2q_3 - q_2q_4 - q_3 + q_4) + p_3p_4(-q_2q_3 + q_3q_4) - p_3q_2q_4 + p_4q_4(q_2 - 1)\right)}{\bar{v}} +$$
$$\frac{S\left(p_1p_3(q_1q_4 - q_2q_4) + p_1p_4(-q_1q_2 + q_1 + q_2q_4 - q_4) + p_3p_4(q_1q_2 - q_1q_4 - q_2 + q_4) + p_3q_4(q_2 - 1) - p_4q_2(q_4 + 1) + p_4(q_4 - 1)\right)}{\bar{v}} +$$
$$\frac{T\left(-p_1p_2(q_1q_4 - q_3q_4) - p_1p_4(-q_1q_3 + q_3q_4) + p_1q_1q_4 - p_2p_4(q_1q_3 - q_1q_4 - q_3 + q_4) - p_2q_4(q_3 + 1) - p_4q_4(q_1 + q_3) - p_4(q_3 + q_4) - q_4\right)}{\bar{v}} +$$
$$\frac{P\left(p_1(p_2(q_1q_2 - q_1 - q_2q_3 + q_3) + p_3(-q_1q_3 + q_2q_3) - q_1(q_2 + 1)) + p_2p_3((-q_1q_2 + q_1q_3 + q_2 - q_3) + (q_3q_2 - q_2 - q_3 - 1))\right)}{\bar{v}} +$$
$$\frac{P\left(p_3(q_1q_2 - q_3q_2 - q_2 - q_3) + q_2 - 1\right)}{\bar{v}} \implies$$

$$u_q(p) = \left( \frac{\begin{matrix} -p_1p_2(q_1 - q_3)(Pq_2 - P - Tq_4) + p_1p_3(q_1 - q_2)(Pq_3 - Sq_4) + p_1p_4(q_1 - q_4)(Sq_2 - S - Tq_3) + p_2p_3(q_2 - q_3)(Pq_1 - P - Rq_4) - \\ p_2p_4(q_3 - q_4)(Rq_2 - R - Tq_1 + T) + p_3p_4(q_2 - q_4)(Rq_3 - Sq_1 + S) + p_1q_1(Pq_2 - P - Tq_4) - p_2(q_3 - 1)(Pq_2 - P - Tq_4) + \\ p_3(-Pq_1q_2 + Pq_2q_3 + Pq_2 - Pq_3 + Rq_2q_4 - Sq_2q_4 + Sq_4) + p_4(-Rq_2q_4 + Rq_4 + Sq_2q_4 - Sq_2 - Sq_4 + S + Tq_1q_4 - Tq_3q_4 + Tq_3 - Tq_4) \\ -Pq_2 + P + Tq_4 \end{matrix}}{\begin{matrix} p_1p_2(q_1q_2 - q_1q_4 - q_1 - q_2q_3 + q_3q_4 + q_3) + p_1p_3(-q_1q_3 + q_1q_4 + q_2q_3 - q_2q_4) + p_1p_4(-q_1q_2 + q_1q_3 + q_1 + q_2q_4 - q_3q_4 - q_4) + \\ p_2p_3(-q_1q_2 + q_1q_3 + q_2q_4 + q_2 - q_3q_4 - q_3) + p_2p_4(-q_1q_3 + q_1q_4 + q_2q_3 - q_2q_4) + p_3p_4(q_1q_2 - q_1q_4 - q_2q_3 - q_2 + q_3q_4 + q_4) + \\ p_1(-q_1q_2 + q_1q_4 + q_1) + p_2(q_2q_3 - q_2 - q_3q_4 - q_3 + q_4 + 1) + p_3(q_1q_2 - q_2q_3 - q_2 + q_3 - q_4) + p_4(-q_1q_4 + q_2 + q_3q_4 - q_3 + q_4 - 1) + \\ q_2 - q_4 - 1 \end{matrix}} \right).$$

Let us consider the numerator of $u_q(p)$. The cross product terms $p_ip_j$ are given by,

$$\begin{gathered} -p_1p_2(q_1 - q_3)(Pq_2 - P - Tq_4) + p_1p_3(q_1 - q_2)(Pq_3 - Sq_4) + p_1p_4(q_1 - q_4)(Sq_2 - S - Tq_3) + \\ p_2p_3(q_2 - q_3)(Pq_1 - P - Rq_4) - p_2p_4(q_3 - q_4)(Rq_2 - R - Tq_1 + T) + p_3p_4(q_2 - q_4)(Rq_3 - Sq_1 + S) \end{gathered}$$

This can be re written in a matrix format given by Equation (1).

$$(p_1, p_2, p_3, p_4)\frac{1}{2}\begin{bmatrix} 0 & -(q_1 - q_3)(Pq_2 - P - Tq_4) & (q_1 - q_2)(Pq_3 - Sq_4) & (q_1 - q_4)(Sq_2 - S - Tq_3) \\ -(q_1 - q_3)(Pq_2 - P - Tq_4) & 0 & (q_2 - q_3)(Pq_1 - P - Rq_4) & -(q_3 - q_4)(Rq_2 - R - Tq_1 + T) \\ (q_1 - q_2)(Pq_3 - Sq_4) & (q_2 - q_3)(Pq_1 - P - Rq_4) & 0 & (q_2 - q_4)(Rq_3 - Sq_1 + S) \\ (q_1 - q_4)(Sq_2 - S - Tq_3) & -(q_3 - q_4)(Rq_2 - R - Tq_1 + T) & (q_2 - q_4)(Rq_3 - Sq_1 + S) & 0 \end{bmatrix}\begin{pmatrix} p_1 \\ p_2 \\ p_3 \\ p_4 \end{pmatrix} \quad (1)$$

Similarly, the linear terms are given by,

$$\begin{gathered} p_1q_1(Pq_2 - P - Tq_4) - p_2(q_3 - 1)(Pq_2 - P - Tq_4) + p_3(-Pq_1q_2 + Pq_2q_3 + Pq_2 - Pq_3 + Rq_2q_4 - Sq_2q_4 + Sq_4) + \\ p_4(-Rq_2q_4 + Rq_4 + Sq_2q_4 - Sq_2 - Sq_4 + S + Tq_1q_4 - Tq_3q_4 + Tq_3 - Tq_4) \end{gathered}$$

and the expression can be written using a matrix format as Equation (2).

$$(p_1, p_2, p_3, p_4)\begin{bmatrix} q_1(Pq_2 - P - Tq_4) \\ -(q_3 - 1)(Pq_2 - P - Tq_4) \\ -Pq_1q_2 + Pq_2q_3 + Pq_2 - Pq_3 + Rq_2q_4 - Sq_2q_4 + Sq_4 \\ -Rq_2q_4 + Rq_4 + Sq_2q_4 - Sq_2 - Sq_4 + S + Tq_1q_4 - Tq_3q_4 + Tq_3 - Tq_4 \end{bmatrix} \quad (2)$$

Finally, the constant term of the numerator, which is obtained by substituting $p = (0, 0, 0, 0)$, is given by Equation (3).

$$-Pq_2 + P + Tq_4 \tag{3}$$

Combining Equation (1), Equation (2) and Equation (3) gives that the numerator of $u_q(p)$ can be written as,

$$\frac{1}{2}p\begin{bmatrix} 0 & -(q_1-q_3)(Pq_2-P-Tq_4) & (q_1-q_2)(Pq_3-Sq_4) & (q_1-q_4)(Sq_2-S-Tq_3) \\ -(q_1-q_3)(Pq_2-P-Tq_4) & 0 & (q_2-q_3)(Pq_1-P-Rq_4) & -(q_3-q_4)(Rq_2-R-Tq_1+T) \\ (q_1-q_2)(Pq_3-Sq_4) & (q_2-q_3)(Pq_1-P-Rq_4) & 0 & (q_2-q_4)(Rq_3-Sq_1+S) \\ (q_1-q_4)(Sq_2-S-Tq_3) & -(q_3-q_4)(Rq_2-R-Tq_1+T) & (q_2-q_4)(Rq_3-Sq_1+S) & 0 \end{bmatrix}p^T + \begin{bmatrix} q_1(Pq_2-P-Tq_4) \\ -(q_3-1)(Pq_2-P-Tq_4) \\ -Pq_1q_2+Pq_2q_3+Pq_2-Pq_3+Rq_2q_4-Sq_2q_4+Sq_4 \\ -Rq_2q_4+Rq_4+Sq_2q_4-Sq_2-Sq_4+S+Tq_1q_4-Tq_3q_4+Tq_3-Tq_4 \end{bmatrix}p - Pq_2 + P + Tq_4$$

and equivalently as,

$$\frac{1}{2}pQp^T + cp + a$$

where $Q \in \mathbb{R}^{4\times4}$ is a square matrix defined by the transition probabilities of the opponent $q_1, q_2, q_3, q_4$ as follows:

$$Q = \begin{bmatrix} 0 & -(q_1-q_3)(Pq_2-P-Tq_4) & (q_1-q_2)(Pq_3-Sq_4) & (q_1-q_4)(Sq_2-S-Tq_3) \\ -(q_1-q_3)(Pq_2-P-Tq_4) & 0 & (q_2-q_3)(Pq_1-P-Rq_4) & -(q_3-q_4)(Rq_2-R-Tq_1+T) \\ (q_1-q_2)(Pq_3-Sq_4) & (q_2-q_3)(Pq_1-P-Rq_4) & 0 & (q_2-q_4)(Rq_3-Sq_1+S) \\ (q_1-q_4)(Sq_2-S-Tq_3) & -(q_3-q_4)(Rq_2-R-Tq_1+T) & (q_2-q_4)(Rq_3-Sq_1+S) & 0 \end{bmatrix},$$

$c \in \mathbb{R}^{4\times1}$ is similarly defined by:

$$c = \begin{bmatrix} q_1(Pq_2-P-Tq_4) \\ -(q_3-1)(Pq_2-P-Tq_4) \\ -Pq_1q_2+Pq_2q_3+Pq_2-Pq_3+Rq_2q_4-Sq_2q_4+Sq_4 \\ -Rq_2q_4+Rq_4+Sq_2q_4-Sq_2-Sq_4+S+Tq_1q_4-Tq_3q_4+Tq_3-Tq_4 \end{bmatrix},$$

and $a = -Pq_2 + P + Tq_4$.

The same process is done for the denominator. □

# 2 Theorem 2 Proof

The optimal behaviour of a memory-one strategy player $p^* \in \mathbb{R}^4_{[0,1]}$ against a set of $N$ opponents $\{q^{(1)}, q^{(2)}, \dots, q^{(N)}\}$ for $q^{(i)} \in \mathbb{R}^4_{[0,1]}$ is given by:

$$p^* = \text{argmax} \sum_{i=1}^{N} u_q(p),\ p \in S_q.$$

The set $S_q$ is defined as all the possible combinations of:

$$S_q = \left\{ p \in \mathbb{R}^4 \;\middle|\; \begin{array}{ll} \bullet \quad p_j \in \{0,1\} & \text{and} \quad \dfrac{d}{dp_k} \displaystyle\sum_{i=1}^{N} u_q^{(i)}(p) = 0 \\ & \text{for all} \quad j \in J \quad \& \quad k \in K \quad \text{for all} \quad J, K \\ & \text{where} \quad J \cap K = \quad \text{and} \quad J \cup K = \{1,2,3,4\}. \\ \bullet \quad p \in \{0,1\}^4 & \end{array} \right\}. \tag{4}$$

*Proof.* The optimisation problem of Equation (5)

$$\begin{aligned} &\max_p : \sum_{i=1}^{N} {u_q}^{(i)}(p) \\ &\text{such that} : p \in \mathbb{R}_{[0,1]} \end{aligned} \tag{5}$$

can be written as:

$$\begin{aligned} &\max_p : \sum_{i=1}^{N} {u_q}^{(i)}(p) \\ &\text{such that} : p_i \leq 1 \text{ for } \in \{1,2,3,4\} \\ &\qquad\qquad -p_i \leq 0 \text{ for } \in \{1,2,3,4\} \end{aligned} \tag{6}$$

The optimisation problem has two inequality constraints and regarding the optimality this means that:

- either the optimum is away from the boundary of the optimization domain, and so the constraints plays no role;
- or the optimum is on the constraint boundary.

Thus, the following three cases must be considered:

**Case 1:** The solution is on the boundary and any of the possible combinations for $p_i \in \{0,1\}$ for $i \in \{1,2,3,4\}$ are candidate optimal solutions.

**Case 2:** The optimum is away from the boundary of the optimization domain and the interior solution $p^*$ necessarily satisfies the condition $\frac{d}{dp} \sum\limits_{i=1}^{N} u_q(p^*) = 0$.

**Case 3:** The optimum is away from the boundary of the optimization domain but some constraints are equalities. The candidate solutions in this case are any combinations of $p_j \in \{0,1\}$ and $\frac{d}{dp_k}\sum_{i=1}^{N} u_q^{(i)}(p) = 0$ forall $j \in J$ & $k \in K$ forall $J, K$ where $J \cap K =$ and $J \cup K = \{1,2,3,4\}$.

Combining cases 1-3 a set of candidate solutions, denoted as $S_q$, is constructed as:

$$S_q = \left\{ p \in \mathbb{R}^4 \;\middle|\; \begin{array}{l} \bullet \quad p_j \in \{0,1\} \quad \text{and} \quad \frac{d}{dp_k}\sum_{i=1}^{N} u_q^{(i)}(p) = 0 \quad \text{for all} \quad j \in J \quad \& \quad k \in K \quad \text{for all} \quad J, K \\ \qquad\qquad\qquad \text{where} \quad J \cap K = \quad \text{and} \quad J \cup K = \{1,2,3,4\}. \\ \bullet \quad p \in \{0,1\}^4 \end{array} \right\}.$$

The derivative of $\sum_{i=1}^{N} u_q^{(i)}(p)$ calculated using the following property (see [1] for details):

$$\frac{dxAx^T}{dx} = 2Ax. \tag{7}$$

Using property (7):

$$\frac{d}{dp}\frac{1}{2}pQp^T + cp + a = pQ + c \text{ and } \frac{d}{dp}\frac{1}{2}p\bar{Q}p^T + \bar{c}p + \bar{a} = p\bar{Q} + \bar{c}. \tag{8}$$

Note that the derivative of $cp$ is $c$ and the constant disappears. Combining these it can be proven that:

$$\begin{aligned} \frac{d}{dp}\sum_{i=1}^{N} u_q^{(i)}(p) &= \sum_{i=1}^{N} \frac{\frac{d}{dp}(\frac{1}{2}pQ^{(i)}p^T + c^{(i)}p + a^{(i)})(\frac{1}{2}p\bar{Q^{(i)}}p^T + \bar{c^{(i)}}p + \bar{a^{(i)}}) - \frac{d}{dp}(\frac{1}{2}p\bar{Q^{(i)}}p^T + \bar{c^{(i)}}p + \bar{a^{(i)}})(\frac{1}{2}pQ^{(i)}p^T + c^{(i)}p + a^{(i)})}{(\frac{1}{2}p\bar{Q^{(i)}}p^T + \bar{c^{(i)}}p + \bar{a^{(i)}})^2} \\ &= \sum_{i=1}^{N} \frac{(pQ^{(i)} + c^{(i)}+)(\frac{1}{2}p\bar{Q^{(i)}}p^T + \bar{c^{(i)}}p + \bar{a^{(i)}})}{(\frac{1}{2}p\bar{Q^{(i)}}p^T + \bar{c^{(i)}}p + \bar{a^{(i)}})^2} - \frac{(p\bar{Q^{(i)}} + \bar{c^{(i)}})(\frac{1}{2}pQ^{(i)}p^T + c^{(i)}p + a^{(i)})}{(\frac{1}{2}p\bar{Q^{(i)}}p^T + \bar{c^{(i)}}p + \bar{a^{(i)}})^2} \end{aligned}$$

For $\frac{d}{dp}\sum_{i=1}^{N} u_q(p)$ to equal zero then:

$$\sum_{i=1}^{N} \left(pQ^{(i)} + c^{(i)}\right)\left(\frac{1}{2}p\bar{Q}^{(i)}p^T + \bar{c}^{(i)}p + \bar{a}^{(i)}\right) - \left(p\bar{Q}^{(i)} + \bar{c}^{(i)}\right)\left(\frac{1}{2}pQ^{(i)}p^T + c^{(i)}p + a^{(i)}\right) = 0, \quad \textit{while} \tag{9}$$

$$\sum_{i=1}^{N} \frac{1}{2}p\bar{Q}^{(i)}p^T + \bar{c}^{(i)}p + \bar{a}^{(i)} \neq 0. \tag{10}$$

The optimal solution to Equation (5) is the point from $S_q$ for which the utility is maximised. □

# 3 Stability of defection

An additional theoretical result that is possible to obtain due to Theorem 1, is a condition for which in an environment of memory-one opponents defection is the stable choice, based only on the coefficients of the opponents.

This result is obtained by evaluating the sign of Equation (4)'s derivative at $p = (0, 0, 0, 0)$. If at that point the derivative is negative, then the utility of a player only decreases if they were to change their behaviour, and thus defection at that point is stable.

**Lemma 1.** *In a tournament of $N$ players $\{q^{(1)}, q^{(2)}, \dots, q^{(N)}\}$ for $q^{(i)} \in \mathbb{R}^4_{[0,1]}$ defection is stable if the transition probabilities of the opponents satisfy conditions Equation 11 and Equation 12.*

$$\sum_{i=1}^{N} (c^{(i)T} \bar{a}^{(i)} - \bar{c}^{(i)T} a^{(i)}) \leq 0 \tag{11}$$

*while,*

$$\sum_{i=1}^{N} \bar{a}^{(i)} \neq 0 \tag{12}$$

*Proof.* For defection to be stable the derivative of the utility at the point $p = (0, 0, 0, 0)$ must be negative.

Substituting $p = (0, 0, 0, 0)$ in,

$$\frac{d}{dp} \sum_{i=1}^{N} {u_q}^{(i)}(p) = \sum_{i=1}^{N} \frac{\left(pQ^{(i)} + c^{(i)}\right)\left(\frac{1}{2}p\bar{Q}^{(i)}p^T + \bar{c}^{(i)}p + \bar{a}^{(i)}\right)}{\left(\frac{1}{2}p\bar{Q}^{(i)}p^T + \bar{c}^{(i)}p + \bar{a}^{(i)}\right)^2} - \frac{\left(p\bar{Q}^{(i)} + \bar{c}^{(i)}\right)\left(\frac{1}{2}pQ^{(i)}p^T + c^{(i)}p + a^{(i)}\right)}{\left(\frac{1}{2}p\bar{Q}^{(i)}p^T + \bar{c}^{(i)}p + \bar{a}^{(i)}\right)^2} \tag{13}$$

gives:

$$\left.\frac{d\sum\limits_{i=1}^{N} {u_q}^{(i)}(p)}{dp}\right|_{p=(0,0,0,0)} = \sum_{i=1}^{N} \frac{(c^{(i)}\bar{a}^{(i)} - \bar{c}^{(i)}a^{(i)})}{(\bar{a}^{(i)})^2} \tag{14}$$

The sign of the numerator $\sum\limits_{i=1}^{N}(c^{(i)}\bar{a}^{(i)} - \bar{c}^{(i)}a^{(i)})$ can vary based on the transition probabilities of the opponents. The denominator can not be negative, and otherwise is always positive. Thus the sign of the derivative is

negative if and only if $\sum_{i=1}^{N}(c^{(i)}\bar{a}^{(i)} - \bar{c}^{(i)}a^{(i)}) \leq 0$. □

A numerical simulation demonstrating the result is given in Figure 1.

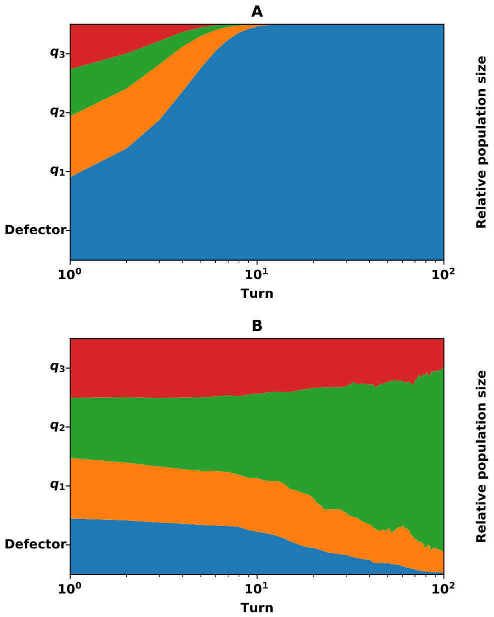


Figure 1: A. For $q_1 = (0.22199, 0.87073, 0.20672, 0.91861)$, $q_2 = (0.48841, 0.61174, 0.76591, 0.51842)$ and $q_3 = (0.2968, 0.18772, 0.08074, 0.73844)$, Equation (11) and Equation (12) hold and Defector takes over the population. B. For $q_1 = (0.96703, 0.54723, 0.97268, 0.71482)$, $q_2 = (0.69773, 0.21609, 0.97627, 0.0062)$ and $q_3 = (0.25298, 0.43479, 0.77938, 0.19769)$, Equation (11) fails and Defector does not take over the population. These results have been obtained by using [2] an open source research framework for the study of the IPD.

## 4 Best response memory-one strategy in environments with noise

Consider an environment where there is a probability $p_n$ that a players actions are executed wrong. This is referred to as the probability of noise. Two memory-one opponents $p \in \mathbb{R}^4_{[0,1]}$ and $q \in \mathbb{R}^4_{[0,1]}$ are now given by:

$$p = (p_1(1 - p_n), p_2(1 - p_n), p_3(1 - p_n), p_4(1 - p_n))$$

and

$$q = (q_1(1 - p_n), q_2(1 - p_n), q_3(1 - p_n), q_4(1 - p_n)).$$

Following a similar approach to that of Theorem 1 it can be shown that the utility $u_q(p)$ is give by:

$$u_q(p) = \frac{\frac{1}{2}pQp^T + cp + a}{\frac{1}{2}p\bar{Q}p^T + \bar{c}p + \bar{a}}, \tag{15}$$

where $Q, \bar{Q} \in \mathbb{R}^{4\times 4}$ are square matrices defined by the transition probabilities of the opponent $q_1, q_2, q_3, q_4$ as follows:

$$Q = \begin{bmatrix} 0 & -p_n^3 (q_1 - q_3)(Pp_nq_2 - P - Tp_nq_4) & p_n^4 (q_1 - q_2)(Pq_3 - Sq_4) & p_n^3 (q_1 - q_4)(Sp_nq_2 - S - Tp_nq_3) \\ -p_n^3 (q_1 - q_3)(Pp_nq_2 - P - Tp_nq_4) & 0 & p_n^3 (q_2 - q_3)(Pp_nq_1 - P - Rp_nq_4) & -p_n^3 (q_3 - q_4)(Rp_nq_2 - R - Tp_nq_1 + T) \\ p_n^4 (q_1 - q_2)(Pq_3 - Sq_4) & p_n^3 (q_2 - q_3)(Pp_nq_1 - P - Rp_nq_4) & 0 & p_n^3 (q_2 - q_4)(Rp_nq_3 - Sp_nq_1 + S) \\ p_n^3 (q_1 - q_4)(Sp_nq_2 - S - Tp_nq_3) & -p_n^3 (q_3 - q_4)(Rp_nq_2 - R - Tp_nq_1 + T) & p_n^3 (q_2 - q_4)(Rp_nq_3 - Sp_nq_1 + S) & 0 \end{bmatrix},$$

$$\bar{Q} = \begin{bmatrix} 0 & -p_n^3 (q_1 - q_3)(p_nq_2 - p_nq_4 - 1) & p_n^4 (q_1 - q_2)(q_3 - q_4) & p_n^3 (q_1 - q_4)(p_nq_2 - p_nq_3 - 1) \\ -p_n^3 (q_1 - q_3)(p_nq_2 - p_nq_4 - 1) & 0 & p_n^3 (q_2 - q_3)(p_nq_1 - p_nq_4 - 1) & p_n^4 (q_1 - q_2)(q_3 - q_4) \\ p_n^4 (q_1 - q_2)(q_3 - q_4) & p_n^3 (q_2 - q_3)(p_nq_1 - p_nq_4 - 1) & 0 & -p_n^3 (q_2 - q_4)(p_nq_1 - p_nq_3 - 1) \\ p_n^3 (q_1 - q_4)(p_nq_2 - p_nq_3 - 1) & p_n^4 (q_1 - q_2)(q_3 - q_4) & -p_n^3 (q_2 - q_4)(p_nq_1 - p_nq_3 - 1) & 0 \end{bmatrix}.$$

$c$ and $\bar{c} \in \mathbb{R}^{4\times 1}$ are similarly defined by:

$$c = \begin{bmatrix} p_n^2 q_1 (Pp_nq_2 - P - Tp_nq_4) \\ -p_n (p_nq_3 - 1)(Pp_nq_2 - P - Tp_nq_4) \\ -p_n^2 (Pp_nq_1q_2 - Pp_nq_2q_3 - Pq_2 + Pq_3 - Rp_nq_2q_4 + Sp_nq_2q_4 - Sq_4) \\ -p_n (Rp_n^2q_2q_4 - Rp_nq_4 - Sp_n^2q_2q_4 + Sp_nq_2 + Sp_nq_4 - S - Tp_n^2q_1q_4 + Tp_n^2q_3q_4 - Tp_nq_3 + Tp_nq_4) \end{bmatrix}, \tag{16}$$

$$\bar{c} = \begin{bmatrix} p_n^2 q_1 (p_nq_2 - p_nq_4 - 1) \\ -p_n (p_nq_3 - 1)(p_nq_2 - p_nq_4 - 1) \\ -p_n^2 (p_nq_1q_2 - p_nq_2q_3 - q_2 + q_3 - q_4) \\ p_n (p_n^2q_1q_4 - p_n^2q_3q_4 - p_nq_2 + p_nq_3 - p_nq_4 + 1) \end{bmatrix}, \tag{17}$$

and the constant terms $a, \bar{a}$ are defined as $a = P + p_n(-Pq_2 + Tq_4)$ and $\bar{a} = p_n(-q_2 + q_4) + 1$.

# References


[1] K. M. Abadir and J. R. Magnus. *Matrix algebra*, volume 1. Cambridge University Press, 2005.

[2] The Axelrod project developers. Axelrod: 4.4.0. *Zenodo* https://doi.org/10.5281/zenodo.1168078, 2016.
